# Combination therapy in patients with acute bipolar mania

**DOI:** 10.1192/j.eurpsy.2023.1473

**Published:** 2023-07-19

**Authors:** M. Gardabbou, R. Feki, I. Gassara, N. Smaoui, S. Omri, M. Maalej Bouali, N. Charfi, L. Zouari, J. Ben Thabet, M. Maalej

**Affiliations:** psychiatry C department, Hedi Chaker, Sfax, Tunisia

## Abstract

**Introduction:**

Numerous guidelines are bending the rule of monotherapy as initial treatment of acute manic episodes and suggest the importance of polytherapy in maximising the treatment efficacy.

**Objectives:**

To assess the polytherapy used in the management of acute manic episodes and the degree of conformity of our prescriptions with international guidelines.

**Methods:**

A retrospective study was carried out for descriptive purposes, targeting the drugs prescribed among patients admitted for the first time for a manic episode within the psychiatry « C » department of Sfax, Tunisia between 2019 and 2022. Patients who received ambulatory care prior to the current episode were excluded.

**Results:**

Our study included 50 male inpatients, with a median age of 31.8 years (min=18, max=62) at the moment of their hospitalisation. Nearly two thirds were single, 82% didn’t get postsecondary education and 65.3% had a profession. The majority (73.5%) belonged to upper-middle class and 67.3% had social security. A quarter of the patients suffered from substance abuse and 14% had a criminal record. Around 89.8% individuals presented a manic episode with psychotic features. The symptoms included mainly heteroaggressiveness in three quarters of cases, agitation in 77.1% and insomnia 76.1%. Insight was good in 79.6% of cases. Polytherapy was preferred to monotherapy in 86% of cases. Bitherapy was used in 74% of cases and tritherapy in only 12%. The most frequent combination was a mood stabilizer plus a second-generation antipsychotic (46%), risperidone plus sodium valproate being used in 34% of cases.

**Conclusions:**

Overall, our prescriptions were in line with the international guidelines and the choice of polytherapy was well argued. Combination therapy is the suggested way to increase treatment efficacy, however, vigilance is required because of the increased risk of side effects.

**Disclosure of Interest:**

None Declared

